# Eye Morphology, Foveal Structure and Photoreceptor Composition in Both Foveae of Common Kestrel (*Falco tinnunculus* Linnaeus, 1758)

**DOI:** 10.3390/biology15120949

**Published:** 2026-06-17

**Authors:** Raúl Cobo, Daniela Jiménez-Díaz, Alicia Navarro-Sempere, Magdalena García, Yolanda Segovia

**Affiliations:** Department of Biotechnology, Faculty of Sciences, University of Alicante, San Vicente del Raspeig, 03690 Alicante, Spain; raul.cobo@ua.es (R.C.); djd1@alu.ua.es (D.J.-D.); alicia.navarro@ua.es (A.N.-S.); m.garcia@ua.es (M.G.)

**Keywords:** *Falco tinnunculus*, retinal layer thickness, bifoveate retina, ocular morphology, pecten oculi, retinal histology

## Abstract

Vision is considered the most important of the five primary senses in birds, particularly in raptors, and the relatively large size of the avian eye reflects its importance. This study provides a morphological and histomorphometric characterisation of the eye and retina of the Common Kestrel (*Falco tinnunculus*), a diurnal raptor with bifoveate retinal organisation. Two adult Common Kestrels, obtained through a wildlife rehabilitation programme, were examined. Eye morphology was characterised using the ratio between corneal diameter and transverse eye diameter, while retinal morphology and layer thickness were evaluated using conventional histological procedures, and opsin expression was examined in both foveae. The eyes showed a globose morphology with a strongly protruding cornea and anterior segment, within the range reported for diurnal birds of prey. Marked regional variation in retinal morphology was observed, with the central retina displaying the greatest overall thickness (254.4 ± 27.04 µm), compared with 108.6 ± 15.58 µm in the peripheral retina. Two distinct foveae were identified: a deep convexiclivate fovea within the area centralis and a temporal fovea with a deep pit and steep walls. Both foveae showed displacement of the inner retinal layers and reduced thickness at the foveal pit. The central and temporal foveae exhibited depths of 217.66 µm and 106.38 µm, respectively. S- and L/M-opsin immunoreactivity was detected in both foveae, and the absence of rhodopsin immunoreactivity in the central foveal pit suggests that high-acuity vision in both foveae is predominantly mediated by cones.

## Simple Summary

Birds of prey rely heavily on vision to hunt, navigate and interact with their environment. The Common Kestrel has two specialised regions in the retina, such as the light-sensitive tissue at the back of the eye, that are believed to provide very sharp vision. In this study, we examined the structure of the eye and retina of the common kestrel and analysed the types of light-detecting cells present in these regions. We found important differences across the retina and identified two specialised pits, called foveae, that are associated with high visual resolution. Both regions contained cells responsible for colour vision and showed little or no evidence of rod photoreceptors, which are mainly used in dim light. These findings improve our understanding of how visual information is processed in birds of prey and provide new information about retinal adaptations linked to their daily activities and hunting behaviour.

## 1. Introduction

Vision represents the primary sensory modality for the survival and ecological adaptation of birds, and in raptors, it reaches a level of anatomical and functional specialisation that exceeds that observed in most terrestrial vertebrates [1,2]. In these species, visual information is essential for locating food resources, navigating complex environments, recognising conspecifics and avoiding predators [2,3]. This functional reliance is reflected in the organisation of the ocular system. In many avian species, the eyes are proportionally larger than those of other vertebrates relative to body mass, and in some cases, approaching the volume of the brain [4,5]. A larger ocular globe increases focal length and allows a larger retinal image to be projected, thereby providing the optical basis for enhanced spatial resolution [6,7]. In addition, the avian visual system includes distinctive structures such as the pecten oculi, a highly vascularised structure arising from the optic disc that has been associated with the metabolic support of the avian avascular retina [8,9].

The avian retina exhibits a remarkable degree of structural diversification, reflecting selective pressures imposed by ecological niche and species-specific behavioural demands [10]. Histologically, the vertebrate retina is organised into a series of well-defined layers, including the photoreceptor layer, outer and inner nuclear layers, outer and inner plexiform layers, and ganglion cell layer, which together process and transmit visual information from photoreceptors to the optic nerve. Compared to the retinas of most other vertebrates, the avian retina is considerably thicker [8]. This increased thickness is attributable to the high density of cells in the inner nuclear layer and the high complexity of neurite arborisation in the inner plexiform layer [11]. These layers are especially thick in the regions with high photoreceptor density, which can be up to twice that of the human fovea [12].

Moreover, a characteristic feature of the avian retina is the presence of areae and visual streaks, high-acuity areae characterised by high photoreceptor densities and large numbers of bipolar and ganglion cells compared to peripheral regions [13]. Apart from areae and visual streaks, many bird species have a third type of retinal specialisation called fovea, a retinal depression in which photoreceptor density reaches its highest values [14,15]. In foveae, the inner retinal layers are displaced centrifugally, reducing light scattering and allowing the image to fall more directly on the outer segments of photoreceptors, thereby enhancing spatial resolution [14,16]. Unlike mammals, in which the fovea is largely restricted to primates, most birds possess one or more foveae whose number, position and depth may vary considerably between species [3,17].

Among birds of prey, retinal organisation varies substantially according to hunting strategy and visual ecology. Diurnal raptors typically possess a bifoveate retina, characterised by the presence of a deep central fovea and a second, shallower temporal fovea [7]. The central fovea, usually deep and funnel-shaped, is associated with lateral monocular scanning and the detection of objects at long distances [14,18]. By contrast, the temporal fovea is oriented towards the frontal binocular visual field and is considered crucial for precise prey fixation and the accurate positioning of the talons during prey capture [19,20]. This bifoveate arrangement enables active predators to maintain high visual acuity along multiple axes of the visual field simultaneously, facilitating both early prey detection and tracking during pursuit [3].

However, this visual configuration is not universal among raptors. Scavenging species such as vultures and condors generally possess a single central fovea and lack a temporal fovea, suggesting a reduced reliance on high-resolution binocular vision during food acquisition [7,18]. Nocturnal raptors, such as owls, typically present a single fovea located in the temporal region of the retina and a rod-dominated retinal organisation that enhances sensitivity under low-light conditions [17]. Taken together, these differences illustrate how sensory ecology and trophic strategy directly shape retinal architecture [19].

The avian retina possesses one of the most advanced cone photoreceptor systems among vertebrates. Light microscopy and spectrometry studies have established that birds have five types of cones, including four single cones that support tetrachromatic colour vision, and a double cone to detect luminance (brightness) and motion. This colour vision is based on cone photoreceptors with maximum sensitivity to long (LWS), medium (MWS), short (SWS) and either violet (VS) or ultraviolet (UVS) wavelengths and a type of long-wavelength-sensitive (LWS) double cone [21,22]. The distribution of different cone types varies across the retina and reflects differences in sensory capacities and in visual ecology [2,3,12,22,23,24,25]. Whereas the retina of nocturnal birds is dominated by rods that increase sensitivity to low light levels, in diurnal birds, cone photoreceptors outnumber rods in all regions of the retina. Moreover, diurnal birds have multiple spectral classes of cones and more complex colour vision: a single type of double cones and several types of single cones, which are characterised by the presence of oil droplets located at the distal end of their inner segments. Diurnal raptors have five types of oil droplets: red, yellow, colourless, green, and pale green. There is a relationship between spectral cone visual pigments and oil droplets, making it possible to classify them [21,22,26]. In many diurnal raptors, the centre of the deep central fovea has been shown to correspond to a rod-free region and, in some species, also to be devoid of double cones, thereby maximising the density of single cones associated with high-resolution vision [5,15]. These characteristics reflect the functional importance of photopic vision in predators that rely on the visual detection of moving prey. In contrast, rods are specialised for vision under low-light conditions and provide high visual sensitivity at the expense of colour discrimination and spatial resolution.

The Common Kestrel represents a particularly valuable model for the study of visual specialisation in birds of prey. This species is diurnal and typically inhabits open landscapes, where it depends heavily on visual cues to detect and track small vertebrates from elevated perches or during hovering flight [5,27]. Although several studies have described general aspects of its ocular morphometry and confirmed the presence of a bifoveate retina, a detailed characterisation of its retinal organisation and high-acuity regions remains incomplete [5,16]. Classical descriptions of the kestrel retina relied primarily on conventional light microscopy, an approach that does not allow precise identification of the different photoreceptor types present within foveal regions [15]. Furthermore, the comparative study of foveal photoreceptor composition across several raptor species conducted by Mitkus and colleagues did not include the Common Kestrel among the taxa examined using opsin immunohistochemical markers [15,24,25].

Although general ocular histomorphometry has been described for Common Kestrel [27], detailed regional morphometric analyses of retinal layers remain scarce. Quantitative descriptions of the structural organisation of the central fovea are still limited. Furthermore, the distribution of photoreceptor opsins within the kestrel fovea has not been specifically examined, leaving important aspects of the cellular basis of high-acuity vision unresolved.

Based on previous descriptions of bifoveate diurnal raptors [15], both foveae were expected to exhibit structural specialisations associated with high-acuity vision and a predominance of cone photoreceptors. In addition, potential differences in morphology and photoreceptor composition between the central and temporal foveae were examined.

In this context, the present study aims to provide a detailed characterisation of ocular morphology and retinal organisation in the Common Kestrel, with particular emphasis on the architecture of the central and temporal fovea. To achieve this, histological analyses, regional retinal morphometric measurements and immunofluorescence using specific opsin markers were combined to examine the distribution of cone subtypes and assess the presence of rods within the region of highest visual acuity. In addition, morphometric analysis of different retinal regions provides an anatomical framework for interpreting the functional specialisation of the fovea in relation to the rest of the retina.

## 2. Materials and Methods

### 2.1. Study Species and Ethical Compliance

This study was based on two adult male Common kestrels (body mass: 161.23 g and 179.56 g) opportunistically obtained through the Santa Faz Wildlife Recovery Centre. The specimens became available through the wildlife rehabilitation programme and corresponded to individuals that had suffered severe traumatic injuries (collisions with power lines and other causes incompatible with successful rehabilitation and release) and were euthanised on veterinary grounds following standard clinical protocols. No gross ocular abnormalities were observed during eye collection and tissue processing. No animals were euthanised specifically for this research. Animals were euthanised by intravenous overdose (500 mg/Kg) of sodium pentobarbital (Eutanax^®^) for clinical reasons unrelated to this study. All procedures complied with European Union Directive 2010/63/EU and Spanish legislation on animal experimentation and were approved by the Ethics Committee of the University of Alicante (Approval no. UA-2020-03-04).

### 2.2. Eye Dimensions

**Figure 1:**
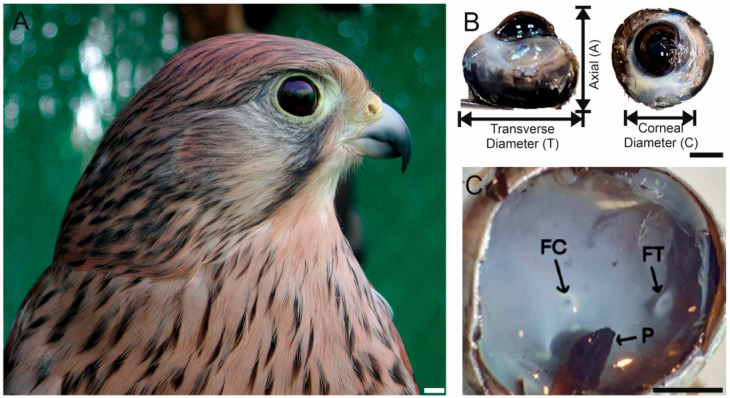
Gross ocular morphology of the Common Kestrel. (**A**) Close-up of the eye. (**B**) Dorsal and side views of excised eyeball showing transverse diameter, axial length and corneal diameter. (**C**) Eyecup showing the temporal fovea (FT), central fovea (FC), and pecten oculi (P). Scale bar: 5 mm.

Immediately after death, the extraocular muscles and connective tissue attached to the posterior aspect of the eyecup were carefully removed. Ocular measurements were obtained using digital callipers (precision ± 0.01 mm). The following parameters (Figure 1B) were recorded for each eye: major globe diameter (T), axial (A) diameter, and corneal diameter (C). Each measurement was repeated three times and averaged for subsequent analysis. Ocular morphometric measurements were obtained from both eyes of each specimen. All morphometric measurements were performed by the same investigator.

### 2.3. Histological Processing for Light Microscopy

After enucleation, the left eyeballs of each specimen were hemisected, and the retinas were examined, oriented and fixed immediately in 4% paraformaldehyde in phosphate-buffered saline (PBS) for 2 h at room temperature. Fixative volume exceeded tissue volume by at least tenfold, following standard histological practice. Histological processing was performed following procedures routinely employed in previous avian retinal studies by our group [28,29] and others [15,30]. After fixation, tissues were dehydrated through a graded ethanol series, cleared in xylene, and embedded in paraffin.

Serial sections (4 μm thickness) were obtained using a Microm HM 340E rotary microtome (Walldorf, Germany) and mounted on gelatine-coated slides. Sections were stained with haematoxylin–eosin following standard histological protocols.

Slides were examined using a Leica DMRB light microscope. Digital images were captured with an Infinity 1 camera at ×20, ×40 and ×100 magnifications under constant illumination settings. Images acquired at ×20 magnification were used for regional retinal assessment and retinal thickness measurements, whereas ×40 and ×100 magnifications were used for detailed evaluation of retinal layers, foveal morphology and photoreceptor organisation. Images were subsequently used for qualitative histological evaluation and morphometric analyses.

### 2.4. Retinal Morphometry

Morphometric analyses were performed on calibrated digital micrographs using Fiji (ImageJ 2). Total retinal thickness and the thickness of each retinal layer were measured in three predefined regions: central retina, peripapillary retina adjacent to the pecten oculi, and peripheral retina. The following layers were evaluated: retinal pigment epithelium (RPE), photoreceptor layer (PRL), outer nuclear layer (ONL), outer plexiform layer (OPL), inner nuclear layer (INL), inner plexiform layer (IPL), ganglion cell layer (GCL), and nerve fibre layer (NFL).

Measurements were obtained from 10 non-overlapping micrographs within each retinal region to minimise local sampling bias. For each micrograph, thickness measurements were repeated three times and averaged prior to analysis. All measurements were performed on images acquired under identical optical conditions to ensure consistency across regions and specimens.

Central and temporal foveae of one eye were identified in sections based on the presence of a well-defined foveal pit and radial displacement of inner retinal layers. Morphometric measurements were obtained from the section exhibiting the maximum foveal depth, which was considered to correspond to the foveal centre. Morphometric parameters included foveal pit depth (c and c’), foveal pit width (d and d’), retinal thickness at the base of the foveal pit (a and a’), and parafoveal retinal thickness (b and b’). Because the foveal measurements were obtained from the section corresponding to the centre of each fovea, these values are presented as representative anatomical measurements rather than as averaged regional estimates.

In addition to quantitative measurements, the structural organisation of the foveal region was qualitatively assessed, including the extent of inner retinal layer displacement and overall laminar configuration within the pit.

### 2.5. Immunolabelling Procedure

Immunofluorescence analyses were performed on unstained paraffin sections containing the central and temporal foveal regions. Sections were deparaffinised, rehydrated, and permeabilised in phosphate-buffered saline (PBS) containing 1% Triton X-100. Antigen retrieval was performed in Tris–EDTA buffer at 90 °C for 20 min, following protocols previously optimised for avian retinal tissue by our group [28,29]. Primary antibodies were applied against S-opsin (AB5407, Chemicon^®^, MilliporeSigma, Temecula, CA, USA, 1:100), L/M-opsin (AB5405, Chemicon^®^, 1:100), and rhodopsin (RET-P1, MAB5316, Chemicon^®^, 1:100). Sections were incubated with primary antibodies for 48 h at 4 °C in a humidified chamber [28,29].

Following thorough washing in PBS, sections were incubated with goat anti-rabbit IgG (H + L) secondary antibody conjugated to CF™568 (SAB4600310, Sigma^®^, Merck/Sigma-Aldrich, St. Louis, MO, USA, 1:500) for 2 h at room temperature. Nuclei were counterstained with DAPI and mounted using Vectashield mounting medium. Negative controls were processed in parallel by omission of the primary antibody to confirm the specificity of immunolabelling.

Fluorescence images were acquired using a Zeiss LSM-800 confocal microscope under identical acquisition parameters for all samples. Image processing and analysis were performed using ZEN Blue 3.0 software without adjustment of relative signal intensity between specimens.

### 2.6. Statistical Analysis

All data are presented as mean ± standard deviation (SD). Retinal layer thicknesses were obtained from the left eyes of two individuals. Given the limited number of biological specimens, analyses were restricted to descriptive statistics, and no inferential statistical comparisons were performed. Data processing and graphical representation were conducted using GraphPad Prism (v9.0).

## 3. Results

### 3.1. Eye Morphology

In the specimens examined, the eye of the Common Kestrel exhibited a globose morphology (Figure 1B). The transverse diameter was 18.97 ± 0.23 mm, the axial diameter 16.26 ± 0.43 mm, and the corneal diameter 9.62 ± 0.64 mm. The axial-to-transverse diameter ratio (A/T) was 0.86, whereas the corneal-to-transverse diameter ratio (C/T) was 0.51. Dissection revealed a well-developed pecten oculi composed of 16 distinct pleats (Figure 1C).

### 3.2. Retinal Organisation and Regional Morphology

**Figure 2:**
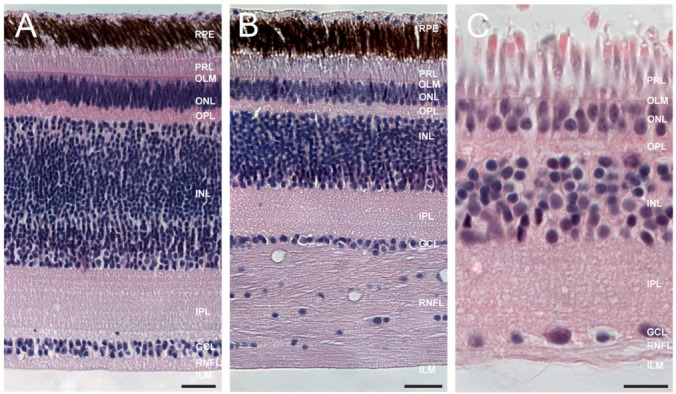
Light micrographs of the retina of the Common Kestrel. Haematoxylin–eosin staining. (**A**) Central retina; (**B**) peripapillary retina adjacent to the pecten oculi; (**C**) peripheral retina. ILM, inner limiting membrane; RNFL, retinal nerve fibre layer; GCL, ganglion cell layer; IPL, inner plexiform layer; INL, inner nuclear layer; OPL, outer plexiform layer; ONL, outer nuclear layer; OLM, outer limiting membrane; PRL, photoreceptor layer; RPE, retinal pigment epithelium. Scale bar = 30 µm.

Histological examination confirmed the typical ten-layered organisation of the vertebrate retina in all analysed regions (Figure 2).

The analysed retinas exhibited a marked regional variation in retinal thickness (Table 1). The central retina exhibited the greatest thickness (254.4 ± 27.04 µm), followed by the peripapillary retina (206.3 ± 33.74 µm), whereas the peripheral retina was substantially thinner (108.6 ± 15.58 µm).

Accordingly, the relative thickness of each layer revealed marked regional variation. The INL was the thickest layer in all regions and decreased from 117.00 ± 17.98 µm in the central retina to 44.27 ± 12.37 µm in the peripapillary region and 31.33 ± 6.80 µm in the peripheral retina. The IPL followed a similar pattern (55.05 ± 6.21 µm centrally; 33.51 ± 3.65 µm peripapillary; 24.12 ± 4.69 µm peripherally).

The GCL was 13.55 ± 2.83 µm in the central retina and decreased towards the periphery (4.11 ± 1.13 µm). In contrast, the NFL reached its maximum thickness in the peripapillary region (78.15 ± 15.91 µm). The ONL and OPL were thicker in the central retina (24.28 ± 3.27 µm and 11.05 ± 1.66 µm, respectively) and progressively thinner towards the periphery. The PRL showed greater thickness in the peripheral retina (23.93 ± 2.66 µm) compared to the central (18.35 ± 4.15 µm) and peripapillary regions (19.85 ± 2.71 µm).

### 3.3. Foveal Structure and Photoreceptor Immunolabelling

**Figure 3:**
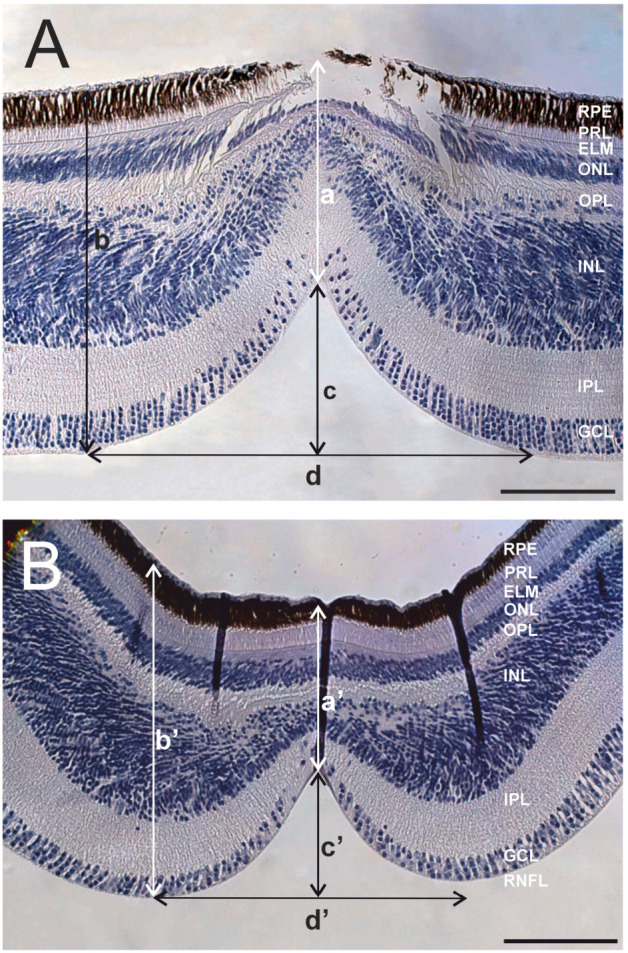
Light micrographs of the foveae of the Common Kestrel. Haematoxylin–eosin staining. (**A**) Central fovea; (**B**) temporal fovea. The morphometric parameters measured are indicated as follows: retinal thickness at the foveal pit (a, a′), parafoveal retinal thickness (b, b′), foveal depth (c, c′), and foveal width (d, d′). Unprimed letters correspond to measurements obtained from the central fovea, whereas primed letters (′) correspond to measurements obtained from the temporal fovea. RPE, retinal pigment epithelium; PRL, photoreceptor layer; ELM, external limiting membrane; ONL, outer nuclear layer; OPL, outer plexiform layer; INL, inner nuclear layer; IPL, inner plexiform layer; GCL, ganglion cell layer; RNFL, retinal nerve fibre layer. Scale bar = 100 µm.

Two distinct foveae were identified (Figure 1C): a central fovea contained within an area centralis and located slightly dorsotemporal to the retinal midpoint, and a temporal fovea, which was characterised by a deep pit with steep walls (Figure 3). Both exhibited radial displacement of the inner retinal layers, forming a funnel-shaped depression. All retinal layers remained identifiable at the foveal pit, although markedly reduced in thickness. The NFL became almost undetectable at the centre of the pit, and reduced cellular density was observed in the ONL, INL, and GCL.

In the central fovea, retinal thickness measured 143.85 µm at the pit and increased to 362.46 µm in the parafoveal region. The foveal depth was 217.66 µm, and the width was 393.08 µm (Figure 3A).

In the temporal fovea, retinal thickness measured 149.46 µm at the pit and increased to 271.01 µm parafoveally. The foveal depth was 106.38 µm, and the width was 265.95 µm (Figure 3B).

**Figure 4:**
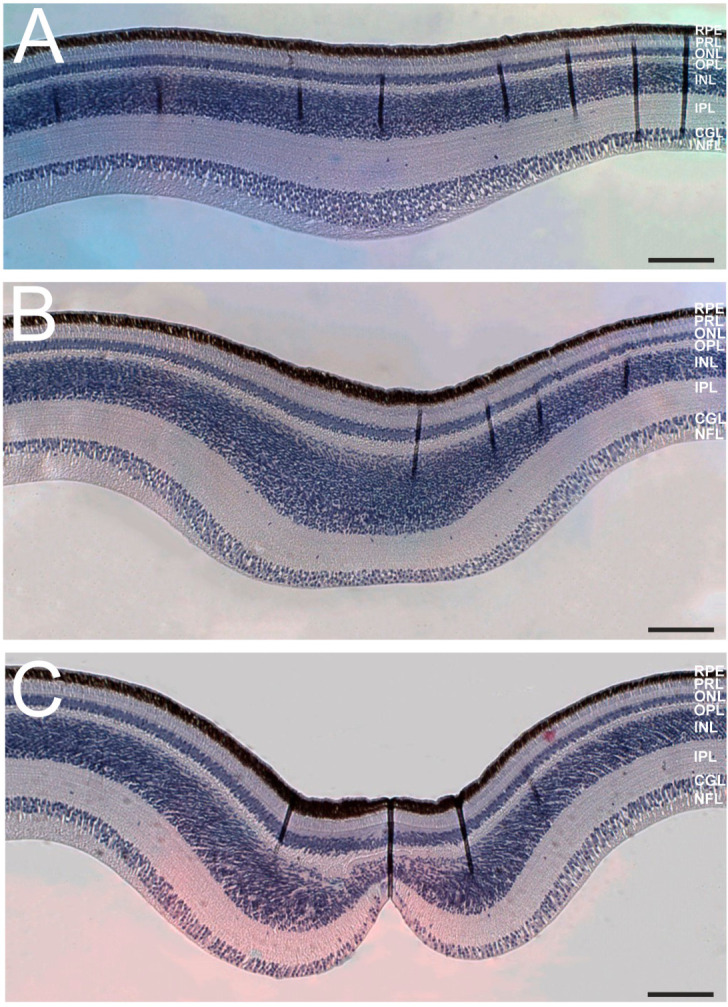
Sequential light micrographs of the temporal foveal region in the Common Kestrel. Haematoxylin–eosin staining. (**A**–**C**) Consecutive sections approaching the temporal fovea. A progressive thickening of the inner retina, particularly the inner nuclear layer (INL), is evident before the appearance of the temporal foveal pit. This thickening becomes increasingly pronounced towards the foveal region and is accompanied by progressive retinal excavation and centrifugal displacement of the inner retinal layers. Scale bar = 100 µm.

An additional structural feature was observed in the retinal region preceding the temporal fovea (Figure 4). Before the foveal pit became evident, the inner retina showed a progressive thickening that became increasingly pronounced towards the fovea, largely due to a marked increase in the thickness of the INL. In the mid-region, a local increase in the GCL and PRL was also observed.

**Figure 5:**
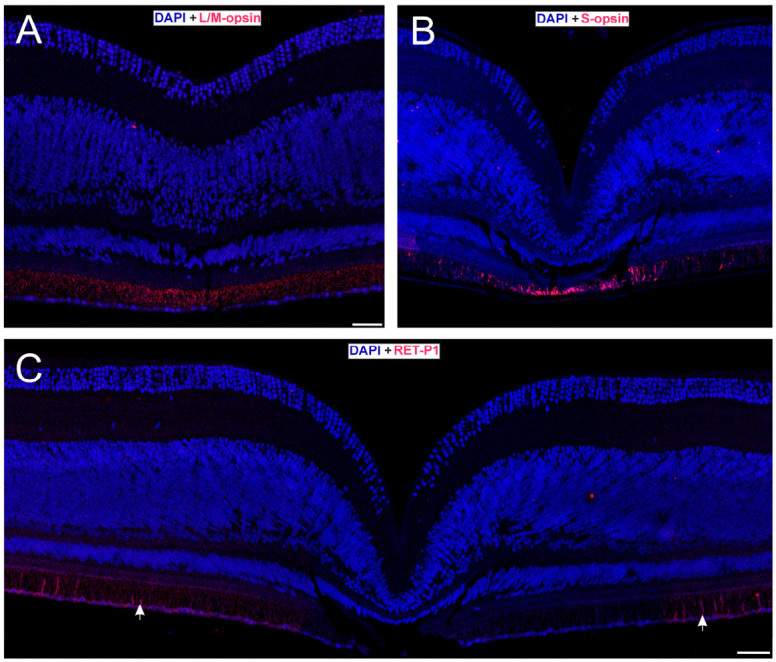
Confocal immunofluorescence labelling of the central foveal region. (**A**) L/M-opsin immunoreactivity showing the distribution of green/red-sensitive cone photoreceptors concentrated within the central fovea. Positive labelling is restricted to cone outer segments, whereas cell nuclei are visualised with DAPI counterstaining (blue). (**B**) S-opsin immunoreactivity showing violet-sensitive cone photoreceptors within the same foveal region. Immunoreactive cone outer segments are visible throughout the foveal photoreceptor layer. (**C**) Rhodopsin (RET-P1) immunolabelling. No rhodopsin immunoreactivity was detected within the foveal pit, consistent with the absence of rod photoreceptors in the central fovea. Arrows indicate rhodopsin-positive photoreceptors located in adjacent extrafoveal retinal regions, providing a positive internal control and facilitating discrimination between specific labelling and background fluorescence. Nuclei were counterstained with DAPI (blue). Images were acquired using confocal microscopy. Scale bar = 50 µm.

**Figure 6:**
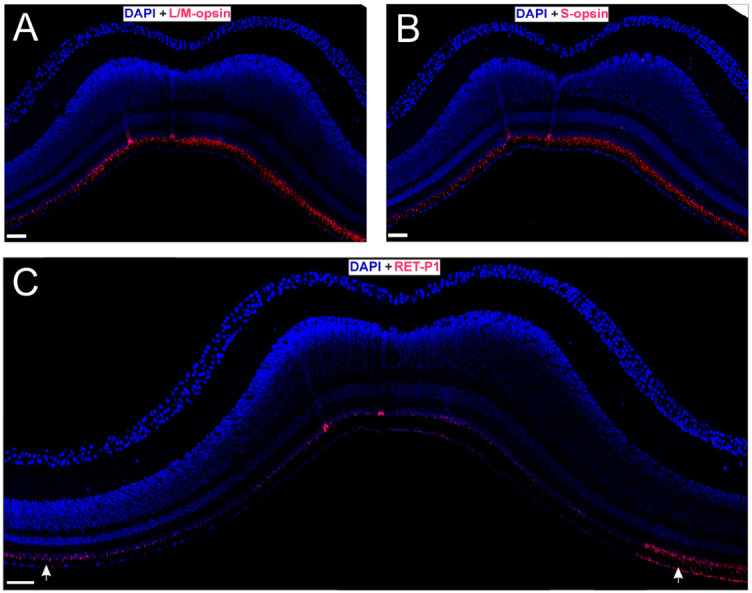
Confocal immunofluorescence labelling of the temporal foveal region. (**A**) L/M-opsin immunoreactivity showing the distribution of green/red-sensitive cone photoreceptors within the temporal fovea. (**B**) S-opsin immunoreactivity showing violet-sensitive cone photoreceptors within the same region. (**C**) Rhodopsin (RET-P1) immunolabelling. No rhodopsin immunoreactivity was detected within the temporal foveal pit, indicating the absence of rod photoreceptors in the foveal region. Arrows indicate representative rhodopsin-positive photoreceptors in adjacent extrafoveal retina, serving as positive internal controls and demonstrating specific immunolabelling outside the fovea. Nuclei were counterstained with DAPI (blue). Images were acquired using confocal microscopy. Scale bar = 50 µm.

Anti-rhodopsin immunolabelling showed no reactivity within the pit of either fovea, although positive labelling was present in adjacent extrafoveal regions. These findings suggest that rods are absent from the pit of both foveae. Immunolabelling for S-opsin and L/M-opsin revealed the presence of violet- and green/red-sensitive cones in both foveae (Figure 5 and Figure 6). Negative controls showed no specific fluorescence signal.

## 4. Discussion

### 4.1. Eye Morphology

Diurnal raptors typically exhibit relatively large ocular globes in relation to body mass, frequently exceeding values predicted for non-raptorial birds [4,31]. The globose eye morphology observed in the Common Kestrel is consistent with classical descriptions of raptor eyes as an optical system specialised for high spatial resolution [4,8]. In the present study, axial length exceeded corneal diameter. Increased axial length has been associated with longer focal distances and larger retinal image size, anatomical features that may contribute to enhanced visual acuity [3,10].

Comparative analyses across vertebrates indicate that the relationship between corneal diameter and axial length is associated with activity patterns [1,32,33,34,35,36,37]. In diurnal animals, relatively smaller diameter corneas regarding axial length limit maximal photon capture but favour increased focal length and image magnification under bright conditions [8,11]. Such a configuration has been proposed to favour visual tasks that require the detection of distant targets under bright illumination conditions, a characteristic commonly associated with diurnal raptors that rely heavily on vision for prey detection and hunting [8,11]. Nevertheless, ocular parameters alone do not determine visual acuity, as additional factors, including corneal refractive power [38] and the density of photoreceptors and ganglion cells [39], also contribute significantly to visual performance. With a cone density in the central fovea of 385,813 cells mm^−2^, Oehme (1964) reported that the Common Kestrel had an anatomical spatial resolution of 59.1 cycles deg^−1^ [40]. Compared with values reported for other raptor species, the spatial resolution of the Common Kestrel is only exceeded by Brown Falcon *Falco berigora*, Indian Vulture *Gyps indicus*, Griffon Vulture, *Gyps fulvus*, Egyptian vulture *Neophron percnopterus* and Wedge-tailed Eagle *Aquila audax* [2]. However, these determinations of spatial resolution are based on behavioural techniques.

The pecten oculi exhibited a pleated morphology with sixteen folds, a value that falls within the range described for diurnal raptors [9,41,42,43,44]. Diurnal species generally possess more complex pectines than nocturnal taxa [8,16], a difference often interpreted as reflecting the metabolic requirements of cone-rich retinas. Comparative studies further suggest that the size, vascularisation and folding pattern of the pecten are associated with the visual ecology and metabolic demands of the avian retina [45,46,47]. In addition, recent work has reported wider and more densely vascularised pectineal vessels in diurnal raptors, potentially supporting the high metabolic demands associated with photopigment recycling and sustained photopic vision [47].

### 4.2. Retinal Organisation and Regional Specialisation

Compared to the retinae of most other vertebrates, the avian retina is considerably thicker [8]. This increased thickness is attributable to the high density of cells in the inner nuclear layer and the high complexity of neurite arborisation in the inner plexiform layer [2,25,48,49]. These layers are especially thick in the regions with high photoreceptor density. The pronounced regional variation in retinal thickness observed in the present study suggests a marked functional specialisation of the Common Kestrel retina. Comparable regional gradients have previously been described in Common Kestrel [27] and in other actively hunting diurnal raptors [7,50]. In these species, increased thickness of the central retina has generally been associated with elevated neuronal densities and enhanced visual processing capacity in regions specialised for high-acuity vision. Similar patterns have been reported in eagles and falcons, where the inner nuclear and plexiform layers contribute substantially to regional retinal thickening. The marked reduction in retinal thickness towards the periphery observed in the present study is therefore consistent with a functional differentiation between high-acuity central regions and peripheral areas primarily involved in visual sensitivity and motion detection. The marked difference between central and peripheral retinal thickness further supports the existence of pronounced regional specialisation within the Common Kestrel retina. Such a gradient highlights the structural specialisation of the central retina as a potential high-acuity zone, consistent with the cone-rich, photopic visual systems characteristic of diurnal raptors [5].

In comparative terms, the central retina of the Common Kestrel (254.4 ± 27.0 µm) is thinner than that reported for several other avian species, including the owl *Otus lettia* (≈300 µm), the pigeon (≈294 µm), the parrot *Amazona aestiva* (≈279 µm), and adult chickens (≈280 µm) [51,52,53,54,55]. In contrast, the peripheral retina of the Common Kestrel is markedly thinner (108.6 ± 15.6 µm), whereas the peripapillary region displays intermediate values (206.3 ± 33.7 µm). Despite differences in absolute retinal thickness among species, the pronounced regional gradient observed in the present study could be consistent with the structural specialisation of the central retina for high-acuity vision commonly described in birds possessing well-developed foveae. These differences may reflect species-specific variation in retinal organisation associated with visual ecology, body size, and behavioural specialisation.

The NFL reached its greatest thickness in the region adjacent to the pecten, likely reflecting the convergence of ganglion cell axons towards the optic nerve head. Previous avian studies have shown that peripapillary regions may contain a higher proportion of myelinated fibres and oligodendrocytes, contributing to increased thickness [56,57]. Vascular structures associated with the pecten may also contribute to this local expansion. The comparatively thinner NFL in the central retina, despite increased GCL thickness, may be explained by the presence of smaller and largely unmyelinated axons in regions associated with high spatial acuity [56].

### 4.3. Foveal Organisation and Photoreceptor Composition

Two distinct foveae were identified: a deep or convexiclivate central fovea with a pronounced invagination, and a similarly deep temporal fovea. Both foveae display a lateral displacement of the inner retinal layers. Dual-foveate retinal organisation is characteristic of actively hunting diurnal raptors [7,15,17,18,58,59,60]. In general, in bifoveate birds, the second temporal fovea is shallow or concaviclivate, consisting of a relatively flat invagination that exhibits little or no lateral displacement of radial fibres [7,14,15,16,18,20,61,62,63,64,65,66]. However, the temporal fovea in Common Kestrel is deep and convexiclivate, although its pit is shallower than that of the central fovea.

Walls (1937) described the deep central fovea as “convexiclivate” and proposed that its steep slopes and refractive index differences could contribute to image magnification [48]. This hypothesis was later partially supported by Locket (1992) [67] and subsequently refined by Snyder and Miller (1978) [68]. The shallower temporal fovea (“concaviclivate”) has been associated with binocular fixation during prey capture [19,52], whereas the central fovea is thought to contribute primarily to long-distance lateral detection [3,20].

Variation in foveal depth and width has been linked to differences in hunting strategy and habitat structure [7,12,17]. The deeper central fovea documented here is therefore consistent with patterns reported in species that pursue mobile prey in open environments and falls within the range described for intermediate to deep avian foveae [14].

An additional structural feature observed in the retinal region preceding the temporal fovea was a progressive thickening of the inner retina, most notably at the level of the inner nuclear layer. This pattern is consistent with the parafoveal rim or shoulder described in avian foveae, which has been associated with centrifugal displacement of inner retinal elements from the foveal centre [14,19]. In raptors, retinal thickness at the edge of the fovea has also been recognised as a relevant structural variable in comparative analyses of foveal morphology [7]. However, to our knowledge, a progressive pre-temporal-foveal thickening of this kind has not been specifically emphasised in previous descriptions of the Common Kestrel retina. In the present material, this thickening was especially evident before the appearance of the temporal foveal pit and was accompanied, in the mid-region, by a local increase in the ganglion cell and photoreceptor layers. Although its exact functional significance cannot be inferred from morphology alone, this thickening could reflect reinforcement of the temporal high-acuity region involved in frontal visual fixation during the final stages of prey capture [19,66]. Immunofluorescence in both the temporal and central foveae revealed S- and L/M-cone opsin immunoreactivity together with the absence of rhodopsin within the foveal pit, suggesting that these regions are predominantly cone-dominated. This organisation is consistent with the cone-rich foveae described in diurnal raptors, where high cone densities and reduced neural convergence have been associated with enhanced spatial resolution under photopic conditions [3,8,13]. In Common Kestrel, a visually guided predator specialised in detecting small terrestrial prey in open habitats, such retinal organisation may contribute to high-acuity vision and chromatic contrast discrimination, as proposed for other diurnal raptors occupying similar ecological niches. Behavioural studies further indicate sensitivity to short wavelengths in kestrels [69,70], whereas molecular evidence suggests violet rather than ultraviolet sensitivity [71]. The reappearance of rhodopsin-positive cells outside the pit likely marks the transition from the foveal region to the more sensitivity-oriented parafoveal retina [15,19,72,73].

## 5. Limitations

The present study was based on a limited number of specimens, and the findings should therefore be interpreted as descriptive anatomical observations. However, studies of ocular morphology and retinal organisation in wild raptors are inherently constrained by the limited availability of suitable biological material. In Spain, the Common Kestrel is included in the *Listado de Especies Silvestres en Régimen de Protección Especial* (Spanish List of Wild Species under Special Protection; LESRPE), and specimens suitable for anatomical investigation are generally only available through wildlife rehabilitation centres or other opportunistic sources. The specimens analysed here became available through a wildlife rehabilitation programme and provided a valuable opportunity to examine the retinal organisation and foveal morphology of this species. Despite these limitations, detailed information on the retinal organisation, foveal morphology and photoreceptor composition of the Common Kestrel remains scarce, and the present study provides novel baseline data that contribute to the comparative understanding of visual specialisations in diurnal raptors.

## 6. Conclusions

The ocular and retinal architecture of the Common Kestrel documented here is consistent with a visual system specialised for high-acuity photopic vision. The globose eye, well-developed pleated pecten oculi, pronounced regional gradients in retinal thickness and the presence of a bifoveate retina collectively reflect the structural adaptations typical of actively hunting diurnal raptors. The deep central fovea, together with the absence of rods within the foveal pit and the strong expression of cone opsins, further support the functional specialisation of this region for enhanced spatial resolution. Despite the limited number of specimens analysed, the present study provides a detailed morphological, histomorphometric and immunohistochemical baseline for the visual system of the Common Kestrel, contributing new data to the comparative understanding of retinal specialisation in Falconidae.

## Figures and Tables

**Table 1:** Thickness of individual retinal layers and total retinal thickness in different retinal regions (μm).

Layer	Central Retina	Peripheral Retina	Peripapillary Retina
NFL	15.34 ± 3.36	5.15 ± 2.50	78.15 ± 15.91
GCL	13.55 ± 2.83	4.11 ± 1.13	6.69 ± 1.68
IPL	55.05 ± 6.21	24.12 ± 4.69	33.51 ± 3.65
INL	117.00 ± 17.98	31.33 ± 6.80	44.27 ± 12.37
OPL	11.05 ± 1.66	7.57 ± 1.23	8.38 ± 1.58
ONL	24.28 ± 3.27	11.89 ± 1.54	15.01 ± 2.49
PRL	18.35 ± 4.15	23.93 ± 2.66	19.85 ± 2.71
Total retinal thickness	254.4 ± 27.04	108.6 ± 15.58	206.3 ± 33.74

Note: Values are expressed as mean ± SD. Measurements were obtained from 10 retinal fields per region within the analysed specimens, and SD reflects the variability of the morphometric measurements used to calculate each regional mean.

## Data Availability

Data supporting the findings of this study are available within the article.

## Abbreviations

The following abbreviations are used in this manuscript:

A	Axial diameter
DAPI	4′,6-diamidino-2-phenylindole
FC	Central fovea
FT	Temporal fovea
GCL	Ganglion cell layer
ILM	Inner limiting membrane
INL	Inner nuclear layer
IPL	Inner plexiform layer
L/M-opsin	Long-/medium-wavelength-sensitive opsin
LWS	Long-wavelength-sensitive cone
MWS	Medium-wavelength-sensitive cone
NFL	Nerve fibre layer
OLM	Outer limiting membrane
ONL	Outer nuclear layer
OPL	Outer plexiform layer
PRL	Photoreceptor layer
RPE	Retinal Pigment Epithelium
S-opsin	Short-wavelength-sensitive opsin
SWS	Short-wavelength-sensitive cone
T	Transverse eye diameter
UVS	Ultraviolet-sensitive cone
VS	Violet-sensitive cone
